# Multicriteria Decision Analysis as a Tool for Assessing Vector-Borne Diseases Risk: The Case of Crimean–Congo Hemorrhagic Fever in Türkiye

**DOI:** 10.3390/microorganisms13091987

**Published:** 2025-08-26

**Authors:** Alessia Milano, Alan Juache, Sarah Houben, Maria Grazia Dente, Claudia Robbiati, Silvia Declich, Ruben Danielyan, Aykut Ozkul, Ilke Karayel-Hacıoglu, Mitra B. Drakulović, Guy Hendrickx, Cedric Marsboom

**Affiliations:** 1 Department of Public Health, Sapienza, University of Rome, Piazzale Aldo Moro 5, 00161 Rome, Italy; 2 Avia-GIS, Risschotlei 33, 2980 Zoersel, Belgium; ajuache@avia-gis.com (A.J.); ghendrickx@avia-gis.com (G.H.); 3 National Center of Global Health, Istituto Superiore di Sanità, Viale Regina Elena 299, 00161 Rome, Italy; mariagrazia.dente@iss.it (M.G.D.); claudia.robbiati@iss.it (C.R.); silvia.declich@iss.it (S.D.); 4 National Center Disease Control and Prevention, Mkhitar Heratsi 12, Yerevan 0025, Armenia; rubendanielyan@yahoo.com; 5 Department of Virology, Faculty of Veterinary Medicine, Ankara University, Şehit Ömer Halisdemir Street, Diskapi, Ankara 06070, Türkiye; aykut.ozkul@ankara.edu.tr (A.O.); ilkeekarayel@gmail.com (I.K.H.); 6 Institute of Public Health of Serbia, dr Subotića Starijeg 5, 112113 Beograd, Serbia; mitra_drakulovic@batut.org.rs; 7 Spatial Epidemiology Laboratory, Université Libre de Bruxelles, 1050 Brussels, Belgium

**Keywords:** risk mapping, multi-criteria decision analysis, vector-borne diseases

## Abstract

Understanding the risk factors for vector-borne diseases, such as Crimean–Congo hemorrhagic fever (CCHF), is critical for effective public health strategies. This study aims to identify and map the environmental and climatic determinants influencing the distribution of *Hyalomma marginatum*, the primary vector of CCHF, in Türkiye, using a Multi-Criteria Decision Analysis (MCDA) approach. A rapid literature review was conducted to identify environmental, climatic, and methodological criteria used in MCDA studies on vector-borne diseases. Semi-structured interviews with local experts from Armenia, Serbia, and Türkiye provided fine-scale data on vector presence. These criteria were analyzed using the Analytic Hierarchy Process (AHP) and combined with Weighted Linear Combination (WLC) within the MCDA framework to produce risk maps for *H. marginatum* occurrence in Türkiye. Key environmental and climatic factors influencing *H. marginatum* distribution, such as temperature, humidity, vegetation, and precipitation, were identified. The MCDA analysis highlighted high-risk regions in Türkiye with elevated suitability for the vector, correlating with areas of human CCHF cases. MCDA risk maps are valuable tools for public health officials, enabling targeted surveillance and interventions. By integrating diverse environmental and climatic variables, this study enhances the understanding of CCHF dynamics and supports the development of focused control strategies.

## 1. Introduction

The intricate interplay between ecological and climatic factors in shaping disease dynamics has long captivated the attention of researchers and policymakers alike [1]. Zoonotic diseases, characterized by their ability to cross species boundaries, can lead to outbreaks in both human and animal populations, disrupting the ecological balance [2]. Effective management and mitigation strategies require a comprehensive understanding of the multifaceted drivers underpinning disease transmission and distribution [3]. In this context, the utilization of the multi-criteria decision analysis (MCDA), a decision-making methodology used to evaluate multiple criteria affecting one multi-layered phenomenon [4], has emerged as a potent tool for unraveling the intricate complexity of disease risk assessment. MCDA has been used in a great variety of disciplines; for example, it has been used in studies involving land suitability [5], environmental research [6], and epidemiological risk assessment [7,8].

The core concept of the MCDA involves identifying a phenomenon of interest and selecting specific variables that contribute to or hinder the occurrence of that event [9,10]. Once the criteria are selected, they are assessed against a ranking and weighted using a predefined scoring function.

MediLabSecure (MLS) is a network whose main goal is to address emerging biological risks in 22 countries in the Middle East, Sahel, Balkans, and Black Sea regions, considering the potential impact of climate and environmental changes on arboviruses such as Rift Valley fever, West Nile, and Crimean–Congo hemorrhagic fever (CCHF) viruses. To achieve this, the project aims to strengthen the capabilities of reference laboratories and public health institutions, while also promoting the implementation of One Health response plans and surveillance at both national and regional levels (https://www.medilabsecure.com) [11]. Of particular importance to this network is the case of CCHF, a viral zoonotic disease primarily transmitted through the bites of infected ticks that can also be transmitted through contact with infected blood and the tissues of animals and humans [12]. The following study was conducted within the framework of this network, which facilitates collaboration among reference laboratories and public health institutions to strengthen surveillance and response capabilities.

The main vector of CCHF is *Hyalomma marginatum*, which exhibits a preference for warm and dry climates and is frequently found in regions characterized by a Mediterranean climate or semi-arid conditions [13]. In Türkiye, CCHF was initially detected in Tokat province in 2002 and has since spread across the nation. From 2002 to 2015, a cumulative total of 9787 cases has been documented, with 469 cases leading to fatalities in humans (4.79% of the total number) [14]. Given this, this work explores the use of MCDA in the risk mapping of vector-borne diseases, based on factors associated with ecological, climatic, and vector-related variables. The primary aims of this study are twofold. The first aim is to assess the risk of the presence and potential distribution of *Hyalomma marginatum*, the main vector of CCHF, in Türkiye by applying an MCDA framework integrating relevant environmental variables. The second aim is to demonstrate the applicability and effectiveness of the combined AHP and Weighted Linear Combination (WLC) methods for risk mapping in complex ecological contexts. First, we conducted a rapid review to examine the application of MCDA in environmental settings to identify the key criteria associated with environmental variables such as the presence of zoonoses and environmental hazards. Along with this literature review, semi-structured interviews with country representatives of Armenia, Serbia, and Türkiye, in the context of MLS, were employed to gather expert information on specific disease transmission drivers. Based on the main factors obtained from the literature review and interviews, we applied a case study approach combining AHP and WLC within an MCDA framework to generate risk maps for the presence of *H. marginatum* in Türkiye.

## 2. Materials and Methods

We implemented an integrated methodological framework structured around three main components: a rapid literature review, expert interviews, and a spatial analysis based on MCDA. The literature review was conducted to identify relevant environmental and climatic variables commonly associated with vector-borne disease transmission, and to examine the methodological approaches most frequently employed in similar studies. The expert interviews provided country-specific insights into the environmental drivers of *H. marginatum* presence and supported the weighting of each criterion in the AHP. Spatial datasets corresponding to the selected variables were then processed to generate risk maps, which were subsequently compared to actual disease occurrence data to assess spatial agreement.

### 2.1. Literature Review

We conducted a rapid literature review, adhering to Cochrane guidance [15], to investigate the application of MCDA in environmental settings in order to identify the key criteria associated with environmental variables such as the presence of zoonoses and environmental hazards. The search for peer-reviewed studies in the literature was performed on Medline via PubMed. The final search string used for the peer-reviewed literature gathering was as follows.

(“MCDM” [Title/Abstract] OR “MCDA” [Title/Abstract] OR “multi criteria decision making” [Title/Abstract] OR “multi criteria decision analysis” [Title/Abstract]) AND “English” [Language] AND 2018/01/01:3000/12/31 [Date—Publication]. The retrieved records were imported into Rayyan software (https://new.rayyan.ai, accessed on 15 March 2024), which facilitated the reading of abstracts and the selection of articles for inclusion or exclusion. The inclusion criteria encompassed: documents detailing the utilization of MCDA methods in contexts where phenomena, processes, or decisions relevant to the natural environment occur; documents published in English with full-text available; documents published between 1 January 2018, and 6 March 2023; and any type of document, including books, reports, and abstracts.

Exclusion criteria included: documents that did not address the research questions and objectives; documents in languages other than English; documents without full-text availability; and documents published before 1 January 2018, or after 6 March 2023.

No formal risk of bias assessment was carried out, as the aim of this review was to identify methodological criteria and decision-making approaches rather than to evaluate the quality or effectiveness of the interventions described in the included studies.

After removing duplicates, we screened the titles and abstracts and performed a full-text screening, adhering to the predefined inclusion criteria. Subsequently, we gathered data concerning, as follows: the specific methodologies of MCDA utilized for determining and combining weights (e.g., Fuzzy Logic, AHP, and WLC, among others); the sectors considered during map development, including environmental, climatic, and physical sectors; and the criteria employed for each sector (e.g., human population density for the human sector, and precipitation for the climatic sector). Moreover, we examined the type of sensitivity analysis performed to evaluate the impact of individual criteria on map creation, as well as the validation method used to validate it. A descriptive analysis was used to summarize the main characteristics of the included studies. For this purpose, we determined the frequency of each method used in each step of the MCDA computing, from core calculations to validation and sensitivity analysis. These findings formed the foundation for generating risk maps in the study’s concluding phase.

To guide the risk mapping procedure, we followed a structured quantitative MCDA approach composed of several key steps:

1. Clearly define and delimit the problem to understand what decision needs to be made such as determining the most probable location for the presence of a disease;
2. Select factors influencing the event based on research or expert evaluation. These may include biological or ecological factors (e.g., presence of vectors), epidemiological indicators (e.g., previous incidence or prevalence rates), demographic data (e.g., population density), and social determinants (e.g., housing conditions, occupational or recreational exposure, and levels of marginalization) relevant to the disease under study;
3. Weight each criterion relative to the others using methods such as the Analytic Hierarchy Model (AHP) method. This involves finding the importance of factors, like prevalence or incidence rates, human population density, or vector density, through a pairwise comparison matrix;
4. Calculate the overall contribution of a factor by combining the factor’s values and their weights. For example, the overall suitability of a disease is determined by combining factors such as prevalence or incidence rates, human population density, and vector density;
5. Deal with uncertainty using techniques like sensitivity analysis, probabilistic modelling, or incorporating error margins into assessments;
6. Review and identify outcomes by assessing the results of decision-making processes and identifying the implications of chosen courses of action [10].

The findings from this review were used to guide both the selection of relevant variables for risk mapping and the definition of a structured MCDA workflow applied in subsequent steps.

### 2.2. Expert Interviews and Variable Selection

Following the rapid review, we conducted semi-structured interviews with representatives from three MLS countries (Türkiye, Serbia, and Armenia) to better understand the drivers associated with the presence of vector-borne diseases in each country. We first asked which diseases were considered a priority for study in their national and regional contexts. We then inquired about the vectors most commonly associated with these diseases and the environmental, climatic, and socio-economic drivers believed to influence vector presence and disease transmission. Participants were asked to provide a ranking of these drivers based on their perceived importance and to specify the conditions under which each driver is most influential. For example, in the case of temperature, they were asked whether vector presence was linked to cold or warm conditions, to identify relevant temperature ranges, and to indicate the most critical seasons for vector activity. Three online meetings were conducted: one with an Armenian representative, one with two representatives from Türkiye, and another with a representative from Serbia. The goal of these interviews was to identify the key drivers associated with the presence of vector-borne diseases, with a particular focus on understanding the role of environmental and climatic factors in the Black Sea and Balkan regions. The four interviewees were domain experts from their respective countries: a medical zoologist from the National Center for Disease Control and Prevention (NCDC) in Armenia; two veterinary virologists from Ankara University in Türkiye; and a public health specialist from the Institute of Public Health in Serbia. Their professional backgrounds ensured the integration of multidisciplinary insights relevant to vector-borne disease dynamics in the region.

Since there is scarce information available for mapping the risk for CCHF, we decided to create risk maps for its main vector, *H. marginatum*, for which adequate information was found. All variables mentioned below were selected based on the literature review results; that is, they were associated with disease transmission. In addition, these variables were consistently mentioned by the representatives. Although a direct mention of the variable was not always obtained (e.g., the representative did not mention that the Normalized Difference Vegetation Index (NDVI) was used to establish disease risk, rather they would mention vegetation presence), proxy variables were employed to perform the analysis. To estimate the risk of *H. marginatum* occurrence we used climatic data including the average temperature, precipitation, and vapor pressure. For environmental data, we employed the NDVI. During the interviews, experts indicated that the presence of the tick is associated with rich vegetation, not directly linked to specific vegetation types. As the included articles in the rapid review used the NDVI as a vegetation index, we decided to adopt it as well. The selected variables reflect a synthesis of the evidence gathered from the literature on environmental drivers of disease incidence and the context-specific validation provided by interviews with national experts. The information gathered from expert interviews was not only used to validate the variables identified in the literature but also served as the basis for assigning weights in the AHP process, reflecting expert perceptions of the relative importance of each driver.

### 2.3. Risk Mapping with MCDA Approach

Based on the variables identified through the literature review and validated by expert input, we retrieved spatial datasets from WorldClim (https://www.worldclim.org, accessed on 16 May 2023) as historical data at the highest resolution (30 s) per month. We selected data from June to August only because this was reported as the period with the maximum presence of *H. marginatum* [13]. We combined the information of individual months into a single dataset by averaging the values. Environmental data, on the other hand, were sourced from Copernicus as historical data at a resolution of 300 m. We normalized the raster layers to a common scale by dividing each value of the raster file by the total sum of the raster values, ensuring that each variable ranged from 0 to 1. We then applied the AHP to establish relative weights for each criterion based on expert knowledge and pairwise comparisons. The AHP process involved creating a pairwise comparison matrix for each criterion and calculating the priority vector to determine the criterion weights. Subsequently, we combined the normalized raster layers using a WLC approach.

To facilitate a comparison between predicted risk and actual incidence, we classified the cumulative number of reported CCHF human cases (2018–2022) at the provincial level into three categories:

− Low: 0 cases;
− Medium: 1–4 cases;
− High: ≥5 cases.

This classification was used to aggregate observed data into interpretable risk levels and support spatial comparison with the predictive risk assessment.

### 2.4. Sensitivity Analysis

Additionally, we conducted a sensitivity analysis using the One-at-a-Time method to understand the map’s sensitivity to variations in the criteria used. For each criterion, we set its weight to zero while keeping the weights of other criteria constant. This enabled us to observe the impact of each criterion on the overall risk map. We calculated the absolute difference between the original risk map and the modified risk map (with one criterion weight set to zero) to quantify sensitivity. These differences were visualized as sensitivity maps, highlighting areas where changes in specific criteria had a significant impact on risk.

### 2.5. Spatial Association with CCHF Cases

A graphical association analysis was performed using disease presence data in the country to assess the association between vector presence and human disease occurrence. For this purpose, we utilized disease presence data collected from 2002 to 2021, provided by Angela Fanelli and Jane Paula Messina [16,17]. We calculated the percentage of georeferenced points located within the risk map areas with scores ranging from 0.7 to 1. This approach enabled us to assess the concentration of points within the risk map areas and provided insights into the levels of risk associated with specific zones. All the maps, validations, and sensitive analyses were conducted in R, version 4.3.2 and QGIS 3.36. This spatial association analysis enabled us to evaluate the real-world applicability of the risk maps developed through the integrated MCDA approach and to identify zones of concordance between the predicted vector presence and observed human disease cases.

## 3. Results

### 3.1. Literature Review Results

**Figure 1:**
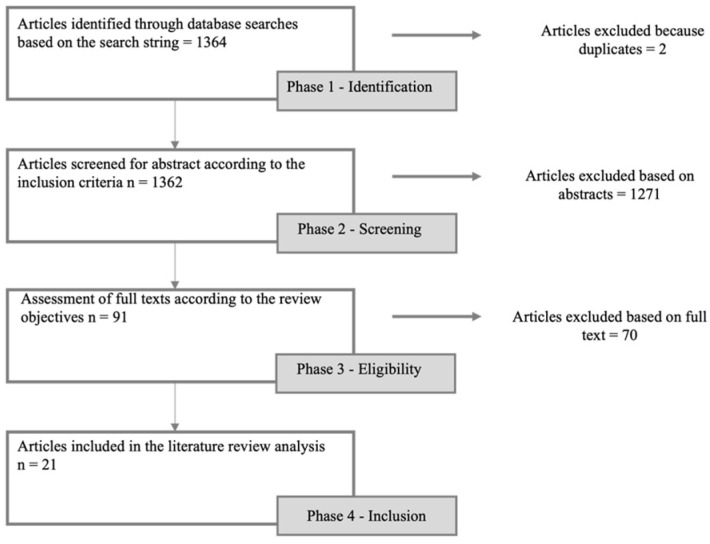
Flow chart summarizing the steps of the rapid review process, from article identification to final inclusion. Horizontal arrows indicate the exclusion of articles at each stage with the corresponding reasons.

The literature search yielded 1364 articles based on the search string. After removing duplicates, 1362 abstracts were screened to determine their relevance and pertinence concerning the research questions. Subsequently, 91 full-text articles were carefully assessed for eligibility based on the inclusion and exclusion criteria. Finally, 21 articles met all the criteria and were included in the rapid review (Figure 1). The theme of the included articles focused on risk maps of diseases affecting animals, both transmissible and non-transmissible to humans, as well as potential environmental disasters.

It was found that 38% (8 out of 21 articles) were dedicated to risk mapping. These articles encompassed various topics: 13 focused on communicable diseases, 2 focused on vectors, 4 focused on natural disasters, and 2 focused on environmental factors. A total of nine articles used a single MCDA method; six used AHP; one used WLC; one used Fuzzy Logic; and another used Simple Additive Expression. Notably, 13 articles employed a combination of methods to create risk maps, with AHP and WLC being the most frequently combined techniques. The literature review also revealed that several criterion types were employed, including domains such as human, animal, vector, ecological, socioeconomic, physical, and climate factors. In Table 1, the list of criteria used in each respective sector is provided.

**Figure 2:**
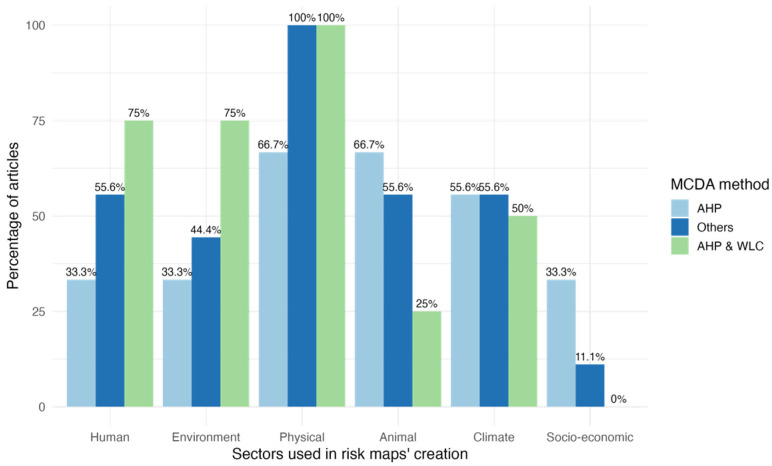
Sectors and specific criteria used in the creation of maps with MCDA.

To highlight the common use of specific methodologies, we grouped AHP and AHP combined with WLC into two categories, while all other methods were combined due to their low representation. Most articles using AHP alone relied on physical and environmental data, with about one-third also incorporating human and climate data. In studies employing AHP with WLC, physical data were universally used, alongside human and environmental data, in the majority of cases (Figure 2).

**Figure 3:**
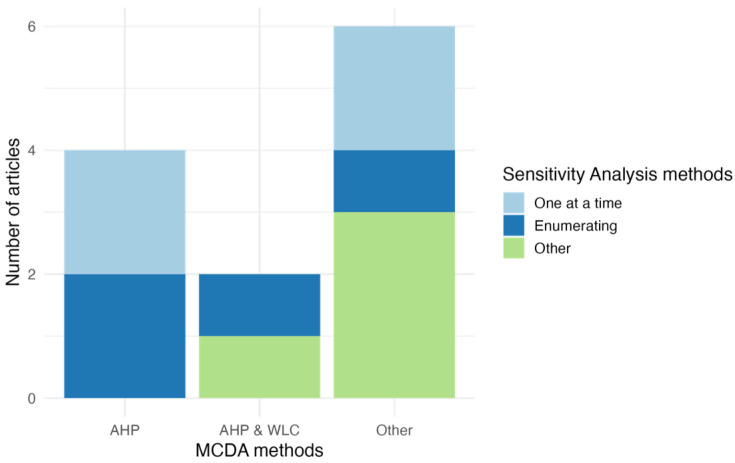
Sensitivity analysis methods used in MCDA articles.

It is worth noting that not all the analyzed articles utilized sensitivity analysis to evaluate the impact of variations in input data on the outcome. However, the predominant sensitivity analysis methods employed were Enumerating and One-at-a-Time. Additionally, four articles utilized different approaches (such as standardization of pairwise comparison matrix, weightless, extensive sensitivity analysis, and weight and membership change), which were categorized as ‘Other’ due to their lack of individual significance, as depicted in Figure 3. This classification was based on their limited representativeness. Furthermore, among the articles that incorporated validation to assess and confirm the accuracy, reliability, and applicability of their methodology, field and cross-validation emerged as the two most frequently used methods.

### 3.2. Interviews

The semi-structured interviews provided complementary insights into the key environmental and climatic drivers associated with the presence of *Hyalomma marginatum* in the Balkans and Black Sea region. The Turkish experts emphasized that the presence of the tick, and consequently the associated CCHF risk, is mainly influenced by medium-to-high temperatures and humidity. The Armenian representative highlighted the relevance of vegetation and precipitation, noting that both contribute to the creation of suitable habitats for tick proliferation. The Serbian expert agreed with the previous assessments and further suggested that livestock presence, human population density, and wildlife could also play a role, although these aspects were not explored in the present study due to data limitations. Taken together, the interviews revealed a clear consensus: temperature and humidity emerged as the most influential environmental factors for tick presence, followed by vegetation cover, and, lastly, precipitation. These four variables were selected from only two of the six commonly used categories (Table 1) of criteria identified in the literature: the climatic category (precipitation, temperature, and vapor pressure) and the ecological/environmental category (NDVI). Other categories, such as physical factors, were excluded due to the lack of a clear link to tick presence, while human and animal-related variables—although highly relevant—were not included due to the unavailability of reliable, high-resolution data for Türkiye.

### 3.3. Crimean–Congo Haemorrhagic Fever in Türkiye

Based on interviews with Turkish representatives, the presence of *H. marginatum* is regarded as the leading factor associated with CCHF risk transmission. The distribution of this tick is linked to elevated temperatures, summer precipitation, vapor pressure, and the presence of vegetation. According to the interviewees, favorable conditions for tick presence occur from June to August, typically when temperatures exceed 22 °C and vapor pressure ranges between 5 and 15 KPa. The availability of vegetation and moderate rainfall during this period were also considered relevant factors.

### 3.4. MCDA for H. marginatum Suitability

**Figure 4:**
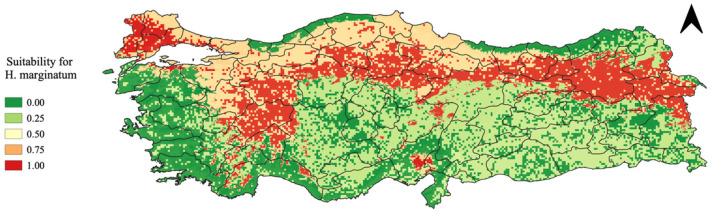
Risk map for *H. marginatum* in Türkiye.

We created a risk map for the distribution of the tick *H. marginatum*, the vector of CCHF, in Türkiye using the MCDA as a framework (Figure 4). The criteria used, ranked from highest to lowest weight, were, as follows: precipitation; NDVI; temperature; and vapor pressure. Table 2 shows the pairwise matrix used to assign scores to criteria for creating the risk map. The values reported in the pairwise comparison matrix were derived from expert responses during the interviews, reflecting the relative importance assigned to each variable.

As shown, the northern part of Türkiye exhibits a notably high risk for the presence of *H. marginatum*, with values near 1. This risk corresponds to areas where the environmental conditions are highly conducive to *H. marginatum*. Conversely, the central-southern regions of Türkiye are depicted as low-risk areas, with values near 0. These regions do not align with the tick’s preferred environmental conditions due to factors such as less suitable temperatures and reduced vegetation cover.

**Figure 5:**
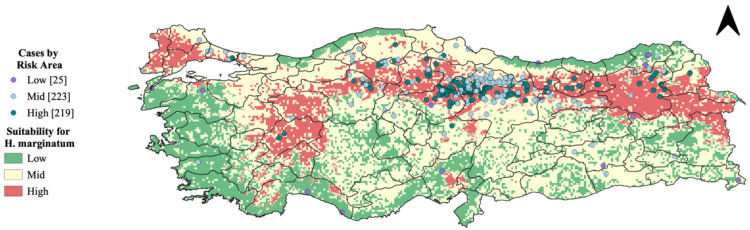
Suitability for *H. marginatum* and CCHF human cases.

**Figure 6:**
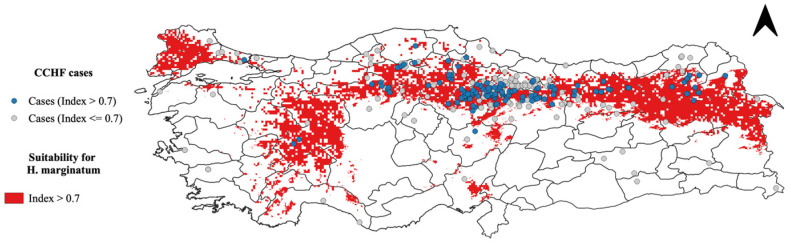
Areas with risk greater than 70% for *H. marginatum* tick presence and reported human CCHF cases.

We overlaid human CCHF disease presence points onto the MCDA output to analyze the spatial relationship between tick presence and human disease cases (Figure 5). Figure 6 highlights regions in red where the probability of encountering *H. marginatum* ticks exceeds 70%. This image aids in understanding the correlation between the high probability of tick presence and the occurrence of human CCHF cases, showing how many of these infections fall within areas where the likelihood of encountering *H. marginatum* is greater than 0.7.

**Figure 7:**
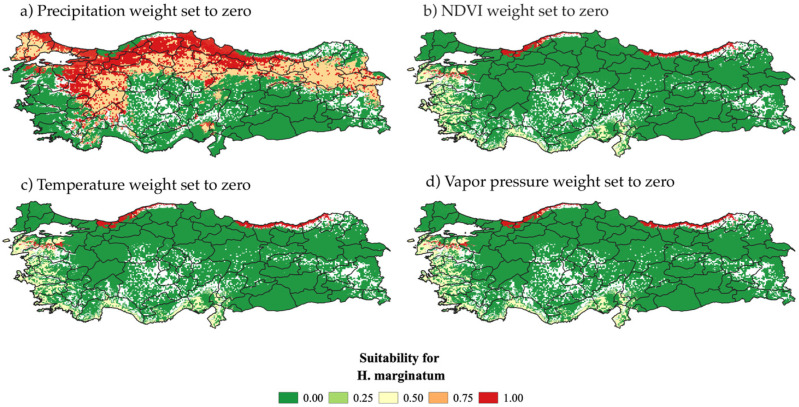
Sensitivity analysis with One-at-a-Time method, showing the effect of excluding each criterion from the model: (**a**) temperature, (**b**) precipitation, (**c**) NDVI, and (**d**) vapor pressure.

To provide a more quantitative assessment of model stability, we performed a One-at-a-Time sensitivity analysis by sequentially setting the weight of each criterion to zero while keeping the others constant. For each modified scenario, we calculated the absolute difference between the original risk map and the modified risk map to quantify the impact of excluding each criterion. These differences were visualized through sensitivity maps, highlighting geographic areas where the model output was most influenced by individual criteria (Figure 7). This quantitative approach allowed us to better understand the relative importance of precipitation, temperature, vapor pressure, and NDVI in shaping the risk landscape.

When the precipitation weight is set to zero (Figure 7a), high-risk patterns persist in northern Türkiye, while central-southern areas shift from low to moderate risk. Setting the NDVI weight to zero (Figure 7b) notably reduces risk in northern Türkiye, shifting some previously low-risk regions to higher risk. Temperature and vapor pressure sensitivity maps (Figure 7c,d) indicate widespread areas of low or zero risk across the study area. The absolute differences between the original risk map and the modified maps highlight the areas most affected by each criterion’s exclusion. The sensitivity maps reveal that precipitation predominantly influences risk levels in the southern and more arid zones, where it is a limiting factor. Conversely, in northern areas with consistently higher rates of precipitation, setting the precipitation weight to zero has less of an effect. Temperature and vapor pressure show stronger influence in northern and central regions, corresponding to their roles in regulating soil moisture and vegetation health. NDVI exhibits a significant impact on delineating high-risk areas overall, with its exclusion causing widespread alterations in risk patterns. This spatial variability underlines the relative importance of each environmental factor and their interaction in shaping the final risk assessment.

To complement the spatial sensitivity maps, we also calculated quantitative indicators of model robustness. These included the absolute difference between maps, the percentage change relative to the original MCDA output, and the coefficient of variation. Additionally, we assessed the total variance explained by each variable’s exclusion. The results confirmed the patterns observed in the spatial analysis. Vapor pressure (72.1%) and temperature (67.9%) accounted for the largest portions of output variance, indicating their strong influence on model stability. NDVI also contributed substantially (45.3%), while precipitation had a more localized effect, reflected in its lower total variance (15.7%). These findings support the observed spatial patterns and reinforce the importance of combining visual and numerical approaches in sensitivity assessment.

## 4. Discussion

The literature review delved into the application of MCDA techniques in environmental contexts, particularly focusing on the criteria used to create risk maps for vector-borne diseases. In summary, AHP and AHP combined with WLC emerged as popular methods for generating risk maps, offering structured criteria prioritization and flexible integration, respectively [18]. Sensitivity analysis, crucial for assessing model robustness, employs Enumerating and One-at-a-Time approaches to scrutinize the impact of criteria variations [19]. Validation methods include field validation, comparing model outputs with real-world data, and cross-validation, ensuring model reliability across different datasets [20].

It must be pointed out that the rapid review process has certain limitations. We focused our search on articles and documents published only between 2018 and 2023 to ensure relevance to current themes; thus, a limited time frame could hinder the number of found results. In other words, this approach led to the exclusion of potentially useful articles published before this timeframe. Furthermore, our selection criteria were restricted to articles discussing the use of MCDA in environmental contexts, narrowing the scope of our research and its findings to this specific area.

Additionally, the use of a single database for the literature search represents a further limitation. While this choice was aligned with the study’s focus on health-related environmental research in the literature and allowed us to efficiently retrieve the most relevant sources, it may have led to the omission of additional studies available in broader multidisciplinary databases. Therefore, the review should be interpreted as a targeted, non-exhaustive synthesis intended to inform the MCDA framework, rather than a comprehensive systematic review.

The semi-structured interviews conducted with representatives from Armenia, Türkiye, and Serbia highlighted the critical role of environmental and climatic drivers in the prevalence of vector-borne diseases in the Balkans and Black Sea region. Temperature, precipitation, and vegetation were frequently cited as key factors influencing disease transmission, both by interviewees and in existing studies in the literature [21,22]. Optimal conditions, such as adequate humidity and temperature, along with rich vegetation, are essential for the survival and reproduction of vectors, thereby increasing the risk of disease transmission. For instance, ticks and mosquitoes, vectors for various diseases, thrive in warm and humid environments [23,24]. Similarly, climate change, which is causing rising temperatures, may further exacerbate the spread of vector-borne disease viruses by creating more favorable conditions for these vectors [25,26].

### 4.1. Principal Findings

The MCDA framework was employed to assess the risk of *H. marginatum*, the vector responsible for CCHF transmission, in Türkiye. *H. marginatum* ticks, vectors of CCHF, thrive in warm and humid conditions [24]. These ticks are known to favor environments characterized by higher temperatures and humidity levels. They are commonly found in regions with warm climates, where they can establish populations and contribute to the transmission of CCHF to humans [25]. Given the limited data available on the presence of CCHF, we focused on mapping the risks associated with the disease vector. Criteria utilized in the analysis, such as precipitation, temperature, vapor pressure, and NDVI, were selected for their relevance to disease transmission, particularly during the period from June to August, when tick activity and human infection risk are highest [24,27]. We chose to apply the AHP in conjunction with WLC to capitalize on the structured decision-making approach provided by AHP’s hierarchical framework, while also benefiting from WLC’s flexibility in integrating diverse criteria into a unified risk assessment. Our decision was further informed by the alignment of our criteria with those used in previous studies employing this combined methodology. Criteria scoring was determined based on interview results with representatives from Türkiye. Consequently, the highest score was assigned to precipitation, followed by NDVI, and, finally, to temperature and vapor pressure, which were given equal weighting.

In the development of risk maps, we solely considered climatic and environmental criteria. This decision stemmed from the unavailability of socio-economic and animal-related data, such as the presence of cattle, sheep, and goat livestock, at a resolution suitable for incorporation into the risk-mapping process. Additionally, data regarding the presence of wildlife were not accessible. These limitations underscore the need for caution when interpreting the results, as certain factors that could potentially influence the risk landscape were not considered due to data constraints.

The generated risk map delineates areas exhibiting varying suitability for the presence of *H. marginatum*, with northern regions demonstrating heightened risk levels in comparison to central-southern areas. This disparity can be attributed to several contributing factors. In northern regions, temperatures during the summer period fall within the tick’s optimal range for activity and reproduction [28]. Moreover, these areas experience sufficient precipitation levels, fostering favorable moisture conditions essential for tick survival and proliferation [29]. The lush, verdant landscapes characteristic of northern regions, as indicated by higher NDVI values, further contribute to the conducive environment by providing the ample vegetation cover conducive to tick habitats [30]. Additionally, the ecological dynamics of these regions, including habitat diversity and wildlife presence, may also play a role in enhancing tick proliferation and persistence, warranting further investigation into the complex interplay between environmental factors and tick distribution patterns [24]. Certain areas identified as being at risk for the presence of *H. marginatum* do not exhibit cases of human infection with CCHF. This discrepancy occurs because the designation of an area as at risk for the tick’s presence does not confirm that the tick is currently there; it merely indicates the potential for its presence based on environmental and ecological parameters. Additionally, the incidence of human disease is affected by numerous factors beyond the mere presence of the tick, including human behavior, livestock management practices, and public health measures. These factors were not included in the map for the reasons previously explained. Consequently, while an area may be suitable for the tick, the lack of reported human infections can be attributed to the complex interplay of various factors that influence the transmission dynamics of CCHF.

### 4.2. Methodological Considerations and Model Limitations

It is important to note that *Hyalomma marginatum* is part of a species complex that includes morphologically similar but ecologically distinct taxa. In this study, we considered the species as a whole, without distinguishing between specific taxa, since all are recognized as competent vectors of CCHF and contribute to the overall disease risk. However, we acknowledge that differences in habitat preferences, host associations, and geographic distribution among these taxa may influence the accuracy of environmental suitability models. Future work could aim to disentangle the distribution patterns of individual members of the complex to provide more taxonomically refined risk assessments.

Combining AHP with WLC offers several advantages to risk mapping. AHP provides a structured approach by breaking down complex decision problems into a hierarchy of criteria and sub-criteria. This facilitates the evaluation of their interdependencies through pairwise comparisons, organizing and rationalizing the decision-making process for a thorough assessment of various risk factors. Furthermore, AHP allows decision-makers to assign weights to criteria based on their relative importance, reflecting their preferences and priorities regarding different risk factors. Integrating decision-makers’ preferences ensures that risk maps accurately reflect their considerations and priorities [31,32]

However, there are also drawbacks to consider. The criteria used in AHP may overlap or be redundant, complicating the decision-making process and leading to ambiguous or suboptimal results [31]. Managing and rationalizing criteria may require thorough analysis and a clarification of relationships [33]. Moreover, the quality and reliability of input data used in AHP–WLC models can significantly influence the results of risk maps [33,34]. Additionally, the assignment of weights to criteria in AHP is subjective and can vary based on the preferences and opinions of experts involved in the decision-making process. This can result in biased or distorted outcomes if expert biases are not adequately managed [31,33,34,35].

The sensitivity analysis conducted on the risk map revealed the varying significance of each criterion on influencing risk levels, highlighting areas where changes in criterion values exerted the greatest impact. While precipitation is a critical factor in environmental risk assessment, particularly in arid regions like Türkiye, its influence diminishes in high-risk zones, where it remains relatively constant. In these areas, temperature, vapor pressure, and NDVI play a more substantial role in delineating risk patterns, especially in northern Türkiye. Fluctuations in temperature and vapor pressure within certain ranges may not substantially affect risk levels, which are also influenced by the geographic and climatic uniformity of the study area.

### 4.3. Future Directions

The findings of this study align with findings in previous research [36,37], indicating the importance of environmental variables in shaping disease risk. The observed patterns of high-risk areas coincide with regions characterized by suitable environmental conditions for vector survival and reproduction. While variations in criteria, such as temperature, precipitation, and NDVI, influence the risk assessment, their relative importance may vary depending on geographic and climatic factors. This study provides a novel contribution by integrating expert-derived knowledge into a structured MCDA framework tailored to the regional context. The inclusion of spatially explicit sensitivity analysis further enhances our understanding of how individual environmental drivers influence risk patterns across Türkiye. Although risk mapping for *Hyalomma marginatum* has been explored in previous studies, high-resolution assessments that combine local expert input with MCDA techniques and sensitivity analysis remain limited. Therefore, the approach presented here offers a context-specific and operationally relevant tool for guiding public health surveillance and vector control strategies. The selection of national experts with diverse professional backgrounds ensured that the identified criteria were grounded in both scientific evidence and country-specific operational knowledge, thereby enhancing the contextual relevance of the risk model.

## 5. Conclusions

This study demonstrates the practical utility of MCDA, combining AHP and WLC, in effectively mapping environmental suitability for *Hyalomma marginatum* in Türkiye, the primary vector of Crimean–Congo Hemorrhagic Fever. The generated risk maps identify key climatic and ecological drivers, providing actionable insights for targeted surveillance and vector control strategies. While limitations related to data availability and subjective weighting remain, the approach offers a flexible and scalable framework adaptable to other vector-borne diseases and regions. Future work should focus on integrating socio-economic and host-related data to enhance risk prediction accuracy and support public health decision-making. In particular, the inclusion of livestock density, land use dynamics, and human mobility patterns could improve the model’s relevance for practical interventions. Additionally, participatory approaches involving local stakeholders in the weighting process may reduce subjectivity and increase model acceptance. Overall, the study highlights the potential of MCDA-based tools to bridge the gap between environmental data and public health planning. By offering a spatially explicit, multi-factorial perspective on vector distribution, this method can support early warning systems, optimize resource allocation, and contribute to integrated vector management strategies. As climate and land use changes continue to alter disease landscapes, such tools will become increasingly important for adaptive and evidence-based health responses.

## Figures and Tables

**Table 1:** Criteria used in each sector.

Sector	Criteria
Human	Density
Animal	Density, distribution
Socio-economic	Knowledge, per capita gross domestic product (PGDP)
Climate	Temperature, precipitation, humidity
Ecological	Habitat characteristics, NDVI, landcover
Physical	Distance from human settlement, distance from nature, soil/water characteristics

**Table 2:** Pairwise comparison matrix used in the AHP analysis, based on expert input from semi-structured interviews.

	Precipitation	NDVI	Temperature	Vapor Pressure
Precipitation	1	2	5	5
NDVI	1/2	1	4	4
Temperature	1/5	1/4	1	1
Vapor pressure	1/5	1/4	1	1

## Data Availability

The original contributions presented in this study are included in the article. Further inquiries can be directed to the corresponding authors.

## Abbreviations

The following abbreviations are used in this manuscript:

MCDA	Multi-criteria decision analysis
AHP	Analytic hierarchy model
MLS	MediLabSecure
CCHF	Crimean–Congo hemorrhagic fever
WLC	Weighted linear combination
NDVI	Normalized difference vegetation index
